# Effects of phloroglucinol on the active phase of labour (EPAL trial): a single blinded randomised controlled trial in a tertiary hospital in sub-Sahara Africa

**DOI:** 10.11604/pamj.2018.30.17.14728

**Published:** 2018-05-09

**Authors:** Charlotte Nguefack Tchente, Theophile Njamen Nana, Paul Nkemtendong Tolefac, Martin Hongieh Abanda, Francky Teddy Endomba Angong, Rita Frinue Tamambang, Gabin Ulrich Kenfack, Georges Nkwelle Mangala, Sagir Muhammad, Marie Solange Doualla, Eugene Priso Belley

**Affiliations:** 1 Faculty of Medicine and Pharmacological Sciences, University of Douala, Cameroon; 2 Service of Obstetrics and Gynaecology, Douala General Hospital, Douala, Cameroon; 3 Faculty of Health Sciences, University of Buea, Cameroon; 4 Faculty of Medicine and Biomedical Sciences, University of Yaoundé 1, Cameroon; 5 Clinical Research Education Networking and Consultancy, Douala, Cameroon

**Keywords:** Phloroglucinol, active labour, duration of labour, rate of cervical dilatation

## Abstract

**Introduction:**

One of the most recognized factors of maternal and neonatal outcome pertaining to the peripartum period is the duration of labour. Finding a drug that will decrease the duration of labour with no effects on mother and foetus will be welcomed. Thereby in this study we aimed to evaluate the effects of phloroglucinol on the duration of the active phase of labour.

**Methods:**

We did a single blinded placebo controlled randomised 1:1 parallel designed superiority trial between January and June 2017 in Douala general hospital. Participants greater than 18 years with singleton uncomplicated pregnancy who consented following randomisation, were administered either 80mg/8ml intravenous phloroglucinol or 8ml of sterile water when in active labour. The primary outcome was the duration of labour. Modified intention to treat analysis was done with the level of significance set at a p value of 0.05.

**Results:**

122 participants received the intervention. The mean total duration labour in the treatment and placebo group were 216.8 ± 38.7 and 358.5 ± 65.8 respectively (p value = 0.243). The mean duration of the active phase of labour in the treatment and placebo group were 183.0±35.6 and 316.0±52.2 respectively (p value = 0.046). The mean rate of cervical dilatation in the treatment and placebo group were 2.1 ± 0.4 and 1.3 ± 0.4 respectively (p value = 0.322). There was no difference in maternal and foetal outcomes between the two groups.

**Conclusion:**

Phloroglucinol shortens the duration of active phase of labour by about 2 hours (42%). It is safe to mother and baby and does not cause adverse foetal or maternal outcomes.

## Introduction

Prolonged labour is associated with multiple adverse maternal and foetal outcomes. The mother is frequently exposed to high risk of infection, obstructed labour, postpartum haemorrhage, perineal injuries, the need for caesarean section and maternal death whereas the foetus faces the danger of infection, asphyxia, various forms of birth injuries and perinatal death [1–3]. O´Driscoll [4] in 1969 at Dublin, introduced the concept of active management of labour and this has over the years influenced the management of the first stage [4]. Active management of labour is associated with a low incidence of prolonged labour and low caesarean section rate [5]. Attempts to accelerate labour without jeopardizing maternal and foetal outcomes is welcomed will be beneficial to the mother, baby and the obstetrician. Over the years several methods have been used to increase uterine contractility and decrease the duration of labour. Some of these methods include amniotomy, use of prostaglandins analogues (misoprostol) and use of oxytocin [6]. These methods, though accelerate cervical dilatation and shorten the duration of labour are often associated with adverse maternal and foetal outcomes such as uterine rupture, postpartum haemorrhage, foetal distress and foetal asphyxia [6]. Antispasmodics may be the solution, an ideal antispasmodic for accelerations of cervical dilations should have a prompt and long lasting action, no adverse effects on uterine contractility and be free from risk of uterine inertia. It should also have minimal side effects on the mother and foetus [7,8]. Antispasmodics frequently used includes phloroglucinol, valethamate bromide, hyoscine butyl-bromide, drotaverine hydrochloride, rociverine and camylofin dihydrochloride [9]. Phloroglucinol is one of antispasmodics, primarily used for gastrointestinal tract colic. The drug was extensively used during 1970s and early 1980s for augmentation of labour [7]. There has been a resurge of interest in the subject. However, there is paucity of data on the use of this drug to accelerate labour. Few studies conducted within the past decade have shown promising results in decreasing the duration of labour and increasing the rate of cervical dilatation [10,11]. To the best of our knowledge, no randomised controlled trial (RCT) regarding the use of this drug and it relationship with the duration of labour have been conducted in sub-Sahara Africa. The only available RCT conducted in Yaoundé, Cameroon by Fouedjio *et al.*evaluated the effects of phoroglucinol on the rate of cervical dilatation and not the duration of labour [11]. The main aim of this study was to investigate the effects of phloroglucionol on the duration of labour and the rate of cervical dilatation.

## Methods

### Study design and setting

The effects of phloroglucinol on the active phase of labour study (EPAL trial) was a prospective single blinded superiority placebo randomised controlled trial in patients admitted in active labour in the maternity of Douala General Hospital (DGH) carried out for five months from January 20^th^ to June 20^th^ 2017. DGH is one of the fastest growing hospitals in the Central Africa sub region. It is a university tertiary teaching hospital located in the economic capital of Cameroon, Douala. Its maternity receives patients in labour including referrals from Douala and its environs. Douala general hospital is well equipped with an imaging centre and clinical laboratory that runs 24/24 where all the necessary investigations can be done.

### Participants

We included all consenting participants in the active phase of uncomplicated labour aged ≥ 18 years with a singleton viable intrauterine pregnancy in cephalic presentation at a gestational age ≥ 37 weeks. We excluded all women with absolute contraindication to vaginal delivery such as double scar uterus, cephalopelvic disproportion (CPD), live threatening bleeding, foetal distress; women with any obstetrical, surgical and severe medical complications such as heart disease and eclampsia; women with multiple pregnancy, preterm labour, preterm premature rupture of membranes; women with known allergies to phloroglucinol.

### Randomisation and masking

Patients were randomly assigned in a 1:1 ratio (parallel design) to receive either the treatment (80mg/8ml phloroglucinol) or the placebo (8ml sterile) based on computer generated codes with a block size of four. Randomisation was done by the use of computer generated codes. The investigators/care givers were aware of the treatment assignment but not the patients. Allocation concealment was ensured, as the randomisation code was not released until the patient has been recruited into the trial, which took place after assessing eligibility and consent.

### Procedures

For patients randomly assigned to the treatment group (group A), they received 80mg/8ml of phloroglucinol at a cervical dilatation of at least 4cm. For patients randomly assigned to the placebo group (group B), they received 8ml of sterile water at cervical dilatation of at least 4cm. Labour progress was monitored with a partogram and a cardiotocogram. All data pertaining to labour events, maternal and neonatal outcomes, and adverse effects of drugs or placebo (nausea, vomiting, blurred vision, dry mouth, and hypotension) were collected. Amount of blood loss after delivery was estimated objectively by weighing the soaked pads. Patients were followed up to 24 hours after delivery.

### Outcome

The primary outcome was the mean duration of labour whereas secondary outcomes included: the rate of cervical dilatation, average blood loss after delivery, early foetal adverse outcome. Other outcomes were adverse effects to the drugs.

### Statistical analysis

The goal of this trial was to established if phloroglucinol was superior to placebo in terms of reduction of the duration of active phase labour and increasing the rate of cervical dilatation and to show the superiority of phloruglucinol in reducing the duration of labour and increasing the rate of cervical dilatation compared to placebo. Using a power of 80% with a 95% confidence interval, from previous studies by Tabassum *et al.* where he had a sample size of 100 participants and reduction in the active phase duration of 66.54 minutes which was clinically significant. Putting all these values in the power formula [12], we estimated the minimum sample size at 141 participants [10]. For maximum recruitment, the study protocol was presented in the service and all obstetricians actively involved. The data was collected with pretested questionnaires using *epi data* software then exported and analysed with SPSS version 20. Percentages and frequencies were determined for categorical variables whereas mean and standard deviations for continuous variables. Chi square test was be used to compare percentages between the two groups and student t test used to compare the mean between the two groups. The level of significance was set at 0.05. Modified intention to treat analysis was used.

### Trial monitoring

A data monitoring committee (DMC) was constituted of three people: a statistician from the clinical research education networking and consultancy (CRENC), principle investigator and one obstetrician and gynaecologist. The DMC was responsible for auditing the trial conduct and this was done twice during the study.

### Ethical consideration

Ethical clearance was obtained from Douala general hospital before starting the trial. Written informed consent was obtained from all participants. The declarations of Helshinki were respected throughout the study period.

**Ethics approval and consent to participate:** ethical approval was obtained from the ethical committee of Douala General Hospital. A copy of is available for review upon request by the Editor-in-Chief of this journal. Written consent for participation was obtained from the patients. Number of the ethics committee approval: **076AR/MINSANTE/HGD/DM/02/17**. **Consent for publication:** consent for publication obtained from the patients. **Availability of data and material:** the data sets supporting the conclusion of this study are available to the editor-in-chief upon reasonable request.

## Results

**Figure 1:**
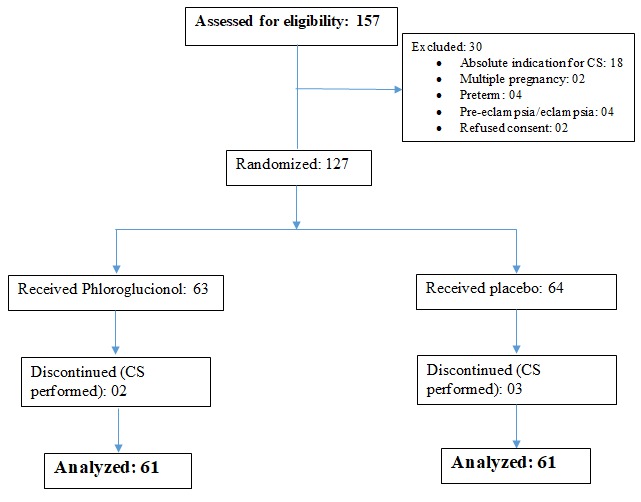
Flow chart showing recruitment and randomisation of participants

We approached 157 participants and assessed them for eligibility, 30 did not meet our inclusion criteria as shown on the flow chart in Figure 1. 127 participants were randomised into two groups: the phoruglucinol group and the placebo group. After randomisation, 5 participants were withdrawn from the trial, a few minute after randomisation they developed foetal distress and caesarean section (CS) was done. The mean ages of the parturients were 26.6±6.1years and 25.8±5.4 years for the treatment group and the placebo group respectively with no statistical significant difference (p value = 0.620). As shown on Table 1, the baseline sociodemographic and obstetric characteristics were the similar in the two groups with no statistical significance difference. The mean gestational age was 38.6±1.2 and 38.7±1.4 in the treatment and placebo group respectively.

**Table 1:** Baseline sociodemographic and obstetric characteristics of the study population

Variable	Treatment group	Placebo group	P value
Mean age (years)	26.6±6.1	25.8±5.4	0.620
Married women – n (%)	42 (68.9%)	37 (60.7%)	0.456
Weight (kilograms)	75.8±4.5	73.2±3.8	0.642
Height (centimetres)	155.2±3.4	155.7±3.1	0.314
Mean gestational age (weeks)	38.6±1.2	38.7±1.4	0.778
Mean cervical dilatation before starting(cm)	4.8±1.1	5.2±1.4	0.124
Augmentation of labour with oxytocin – n (%)	42 (68.9%)	47 (77.0%)	0.576
Parity			
Parity - Mean±SD	2.2±0.1	2.6±0.2	0.543
Primipara- n (%)	13 (21.3%)	9 (14.7%)	0.062
Multipara – n (%)	44 (72.1%)	45 (73.8%)	0.567
Grand multipara – n (%)	4 (6.6%)	7 (11.5%)	0.092

### Primary outcome

The mean total duration of labour was lower in the treatment group compared to the placebo group, however this was not statistically significant as shown on Table 2. There was a mean difference of 133 minutes (2.2 hours) in the duration of the active phase of labour between the treatment group and the placebo group that is the mean duration of the active phase of labour was 42% shorter compared to the placebo. This was statistically significant (p value = 0.046).

**Table 2:** Mean duration of the different stages of labour

Variable	Treatment group	Placebo group	P value
First stage active phase (minutes)	183.0±35.6	316.0±52.2	0.046
Second stage (minutes)	25.1±6.2	34.5±5.5	0.322
Third stage (minutes)	8.7±3.4	11.1±2.0	0.082
Total duration (minutes)	216.8±38.9	358.5±65.8	0.243

### Secondary outcomes

The mean rate of cervical dilatation was 2.1 ± 0.4cm/hour and 1.3 ± 0.4cm/hour for the treatment and placebo group respectively with a difference in the rate of cervical dilatation of 0.8cm/h between the treatment and the placebo group. This was however not statistically significant (p value = 0.322). There was no statistically significant difference between the average blood losses in the two groups as shown on Table 3. For neonatal outcome, there was no statistically significant difference between the two groups in the Apgar score at the first and fifth minute. We registered one case of intrapartum foetal death (1.6% mortality rate) in the phloroglucinol group compared to none in the placebo group. This was not statistically significant. Similarly, we had three cases of neonatal asphyxia, one (1.6%) in the treatment group and two (3.2%) in the placebo group. This was not statistically significant. We had one case of maternal adverse effect in the treatment group, this was not statistically significant. The particular maternal adverse effect in the treatment group was vomiting which occurred three minutes following injection of the product. There was no case of blurred vision, dry mouth, hypotension and allergy.

**Table 3:** Maternal and foetal outcomes and adverse effects of phloroglucinol

Variable	Treatment group	Placebo group	P value
Maternal outcome			
Average blood loss (millilitres)	405.5±72.9	426.0±62.5	0.052
Mean rate of cervical dilatation (cm/hour)	2.1±0.4	1.3±0.4	0.322
Maternal adverse effects-n (%)	1 (1.6%)	0 (0.0%)	0.084
Neonatal outcome			
Apgar at first minute	8.70±0.4	8.3±0.7	0.246
Apgar at fifth minute	9.7±0.2	9.1±0.4	0.067
Intrapartum foetal death- n (%)	1 (1.6%)	0(0.0%)	0.324
Neonatal asphyxia- n (%)	1 (1.6%)	2 (3.2%)	0.089

## Discussion

In modern obstetrics, a drug that shortens the duration of labour without jeopardizing maternal and foetal outcomes is welcome both by the obstetrician and the woman. The cervix usually dilates at a rate of 1-2cm [13]. Phloroglucinol is thought to facilitate labour by reducing spasms and oedema of the cervix, harmonize shrinkage of the uterus, all these with no effects on uterine contractions. Since 1960 research indicated that phloroglucinol could be improve cervical dilatation especially in the active phase of labour [9].

In the EPAL trial, the authors investigated the effects of phloroglucinol, a spasmolytic, on labour. We found out that the mean total duration of labour (first, second and third stage combined) was about 3.6 hours in the phoroglucinol group compared to about 6 hours in the placebo group. The difference in the total duration of labour of about 2.4 hours was not statistically significant. This means that the delivery in women without Phloroglucinol was completed about 2.4 hours later compared to women with phloroglucinol. This is similar to studies done earlier on phloroglucinol by Tabussum *et al.* where they had 2 hours mean difference between the two groups [10]. Studies done on other spasmolytic such as hyoscine has a difference of about one hour in the total mean duration of labour [14]. Similarly, the duration of active phase labour was about 2.2 hours lower in the ploroglucinol group compared to the placebo group that is the mean duration of the active phase of labour was 42% shorter in the treatment group compared to the placebo. This was statistically significant with a p value of 0.046. This is equally similar to studies done on phloroglucinol as well as other spasmolytic [10,14]. The cervix dilated 0.8cm/hour faster in the phloroglucinol group compared to the placebo. This was however not statistically significant (p value = 0.322). This is similar to studies done in Cameroon by Fouedjio et al [11] where the rate of cervical dilatation was about 0.5cm/hour faster in the phloroglucinol group compared to the treatment group and to studies conducted in other developing countries where the rate was about 0.8cm/hour in favour of the phloroglucinol group [10]. This translates and confirms the assertion that labour is faster in parturient with phloroglucinol compared to those without phloroglucinol as the faster the rate of cervical dilatation, the faster the labour.

There was no statistical significant difference between the mean Apgar scores at the first and fifth minute. Similarly, no difference in adverse foetal outcomes like intrapartum foetal death and asphyxia. Though we registered one case of intrapartum foetal death in the phloroglucinol group and three cases of neonatal asphyxia (one in the phloroglucinol group and two in the placebo group), this was not statistically significant. This go to confirm that phloroglucinol has no effect on the foetus. This had earlier been confirmed by similar studies [10,11]. The main limitation of the present study was that we included only healthy singleton pregnancies and that the study was a single blinded and carried out in a single centre, the investigators/data collectors could be influenced by the fact that they were aware of the participants on phloroglucinol and those on placebo. However, our results can serve as reference and a pilot study for future large multicentre studies. We therefore recommend that larger multicentre randomised controlled trials be carried to ascertain the efficacy of this drug on the duration of labour.

## Conclusion

A single dose of 80 mg of intravenous phloroglucinol is both an effective and safe means of decreasing up to two hours the duration of the active phase of labour in singleton uncomplicated cephalic pregnancies without any adverse effects on the foetus or mother. Further studies are needed to evaluate the effectiveness of this drug on the total duration of labour and the rate of cervical dilatation and effacement as well as on pregnancy with complications. We recommend a large multicentre RCT to be carried out.

### What is known about this topic

- Phloroglucinol may reduce the duration of labour;
- Phloroglucinol may increases the rate of cervical dilatation.

### What this study adds

- Single dose of 80mg phloroglucinol decreases the duration of the active phase of the first stage of labour by about 2 hours;
- Phloroglucinol is safe to both mother and foetus.
